# Implantable acousto-optic window for monitoring ultrasound-mediated neuromodulation *in vivo*

**DOI:** 10.1117/1.NPh.9.3.032203

**Published:** 2022-07-20

**Authors:** Sungho Lee, Keunhyung Lee, Myunghwan Choi, Jinhyoung Park

**Affiliations:** a Seoul National University, School of Biological Sciences, Seoul, Republic of Korea; b Seoul National University, Institute of Molecular Biology and Genetics, Seoul, Republic of Korea; c Sungkyunkwan University, Department of Intelligent Precision Healthcare Convergence, Suwon, Republic of Korea; d Sungkyunkwan University, Department of Biomedical Engineering, Suwon, Republic of Korea

**Keywords:** ultrasound, neuromodulation, *in vivo* two-photon

## Abstract

**Significance:**

Ultrasound has recently received considerable attention in neuroscience because it provides noninvasive control of deep brain activity. Although the feasibility of ultrasound stimulation has been reported in preclinical and clinical settings, its mechanistic understanding remains limited. While optical microscopy has become the “gold standard” tool for investigating population-level neural functions *in vivo*, its application for ultrasound neuromodulation has been technically challenging, as most conventional ultrasonic transducers are not designed to be compatible with optical microscopy.

**Aim:**

We aimed to develop a transparent acoustic transducer based on a glass coverslip called the acousto-optic window (AOW), which simultaneously provides ultrasound neuromodulation and microscopic monitoring of neural responses *in vivo*.

**Approach:**

The AOW was fabricated by the serial deposition of transparent acoustic stacks on a circular glass coverslip, comprising a piezoelectric material, polyvinylidene fluoride-trifluoroethylene, and indium-tin-oxide electrodes. The fabricated AOW was implanted into a transgenic neural-activity reporter mouse after open craniotomy. Two-photon microscopy was used to observe neuronal activity in response to ultrasonic stimulation through the AOW.

**Results:**

The AOW allowed microscopic imaging of calcium activity in cortical neurons in response to ultrasound stimulation. The optical transparency was ∼40% over the visible and near-infrared spectra, and the ultrasonic pressure was 0.035 MPa at 10 MHz corresponding to $10\;\mathrm{mW}/{\mathrm{cm}}^{2}$. In anesthetized Gad2-GCaMP6-tdTomato mice, we observed robust ultrasound-evoked activation of inhibitory cortical neurons at depths up to 200  μm.

**Conclusions:**

The AOW is an implantable ultrasonic transducer that is broadly compatible with optical imaging modalities. The AOW will facilitate our understanding of ultrasound neuromodulation *in vivo*.

## 1 Introduction

Neuromodulation techniques are indispensable for investigating neural circuit functions and for treating neurological diseases.^1^^–^^3^ Neuromodulation is typically mediated by intracranial delivery of different forms of energy, including electric (e.g., transcranial direct-current stimulation and deep brain stimulation),^4^^–^^6^ magnetic (e.g., transcranial magnetic stimulation)^7^ waves, and light (e.g., optogenetics). As an alternative form of energy, ultrasound-mediated neuromodulation has emerged as a promising tool because it simultaneously provides high spatiotemporal precision and deep soft-tissue penetration without requiring any genetic or surgical perturbation. The efficacy of ultrasound-mediated neuromodulation has been repeatedly demonstrated in experimental small animals, such as triggering tail flickering or whisker movement by stimulating the motor cortex and ameliorating the epileptic phenotype by subcortical stimulation.^8^^,^^9^ Ultrasound neuromodulation is also effective in triggering antisaccadic eye movement in non-human primates and boosting sensory performance in human subjects.^10^^,^^11^ In contrast, our understanding of the neuromodulatory mechanism of ultrasonic waves is limited; potential molecular candidates range from cell membranes to ion channel.^12^ Adding more complexity, ultrasound has a wide range of parameters, including frequency, duration, and duty cycle, which potentially lead to different biological outcomes. The brain is also composed of multiple neuronal and glial subtypes, complicating the generalized understanding.

Two-photon microscopic imaging on a cranial window model is a well-established approach to observe spatiotemporal activities of specific neuronal and glial subtypes longitudinally *in vivo*. However, most conventional ultrasound transducers are barely compatible with microscopy systems. First, the form factors of conventional ultrasound transducers are typically large compared to the several millimeter-scale working distances of a typical high numerical aperture objective lens.^13^^,^^14^ Second, the glass coverslip used in the cranial window model largely prevents the delivery of ultrasonic waves due to high acoustic impedance, which compromises target specificity by transforming the longitudinal wave into the shear wave.^15^^,^^16^ Consequently, observing functional activities of neuronal cells under controlled ultrasound stimulation is technically challenging.

Here, we propose a solution to this technical challenge by introducing an optically transparent acoustic transducer, termed the acousto-optic window (AOW). The AOW simultaneously allows ultrasound stimulation over a defined brain area and two-photon fluorescence imaging of neuronal activity through the transducer. We describe the fabrication of an AOW based on a transparent piezoelectric material and its surgical implantation on a mouse brain under anesthesia and demonstrate the feasibility of recording ultrasound-evoked functional activity in a genetically specified neuronal subtype *in vivo* using two-photon microscopy.

## 2 Materials and Methods

### 2.1 AOW Fabrication

**Fig. 1:**
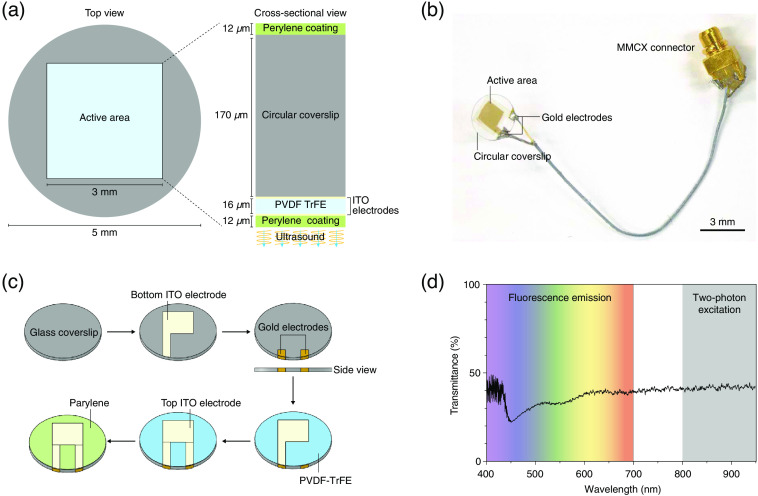
The AOW. (a) Schematic layout of the AOW. The AOW is fabricated on a circular glass coverslip with a diameter of 5 mm. The squared active area contains a piezoelectric material (PVDF-TrFE) and ITO electrodes for ultrasound generation. (b) Photograph of the fabricated AOW. (c) A step-by-step process for fabricating AOW. (d) Optical transmittance of the AOW active area. The visible spectral region (400 to 700 nm) was used for collecting fluorescence emission. The near-infrared spectral region (800 to 1000 nm) was used for two-photon excitation of a femtosecond laser.

Surface of glass coverslip was cleansed using a series of solutions of acetone (67-64-1, Samchun Pure Chemical), degenerative alcohol (64-17-5, Samchun Pure Chemical), 1% water dissolved detergent (1104-1, Alconox), and deionized water. After masking the glass coverslip with a Kapton masking tape (CT-10, iNexus) excluding the central region ($∼3\times 3\;{\mathrm{mm}}^{2}$), it was cleansed with an oxygen plasma cleaner (PDC-002-HP, Harrick Plasma) for 20 min at 20 sccm as shown in Fig. 1(a). An indium–tin–oxide (ITO) electrode (${\mathrm{In}}_{2}O_{2}:{\mathrm{Sn}}_{2}O_{2}$, 9:1 wt/wt) was loaded onto the glass coverslip using a DC magnetron sputtering system (Flexlab-50, A-Tech System) to form an optically transparent bottom electrode. The ITO-coated coverslip was heated up to 300°C on a hot plate for 1 h to increase the transparency and conductivity.^17^ Because the cable was soldered on the other side of the transducer, the coverslip was flipped, and two gold island electrodes for the cable connection were formed by sputtering a gold electrode extended to the edge of the coverslip to make connections to the ITO electrode. The polyvinylidene fluoride-trifluoroethylene (PVDF-TrFE) solution was prepared by dissolving 10 g of PVDF-TrFE powder (FC-20, Piezotech) in 15 g dimethylacetamide (271012, Sigma-Aldrich) and 36 g methyl ethyl ketone (63140-1230, Junsei). The solution was spin-coated on the ITO-loaded coverslip at 500 rpm for 30 s to obtain a uniform layer of 16  μm thickness. The sample was cured in a dry oven at 70°C for 30 min and further baked at 135°C for 2 h. The top electrode was also formed on the PVDF-TrFE layer by sputtering ITO, followed by masking the patterned electrode and roughening the surface with oxygen plasma to enhance the adhesion of ITO on the polymeric surface. A 38-μm-thick coaxial cable (38 AWG micro coax cable, Samtec) was bonded on the prepared gold electrode with the soldering temperature of 280°C. To obtain the piezoelectric characteristics of PVDF-TrFE, the application of a high DC voltage of 100  MV/m along the transducer, called poling process, was performed in a dry oven at 105°C for 20 min. Subsequently, a layer of parylene (Parylene-N, Suzhou Chireach Biomedical Technology Co., Ltd.) was created on the transducer using a parylene coater (OBT-PC200, Obang Technology) to form an acoustic matching and waterproof layer. The AOW was connected to the Micro-Miniature Coaxial (MMCX) connector to prevent gnawing of the cable by the mouse. The cable length was maintained as short as 5 cm, and an MMCX connector (C-RMMF. WST0.1, Ace RF Comm) with a small form factor was selected [Figs. 1(b) and 1(c)].

### 2.2 Measurement of Acoustic Performance of the AOW

The AOW acoustic performance was characterized by measuring its pulse-echo response, surface pressure distribution, and acoustic beam profile. The pulse-echo test was conducted using a pulser/receiver (UT 340, UTEX Scientific) with an electrical transmit pulse of 1 ns of $100\;V_{\mathrm{pp}}$ and a gain of 20 dB. During the test, the transducer was placed at 12 mm above a flat crystal reflector with its natural focus.

The acoustic beam profile of the AOW was measured using a needle hydrophone (NH1000, Precision Acoustics) in a tank filled with deionized water. The needle hydrophone was installed in a custom-made motorized three-axes stage. The beam profile of the AOW was scanned with a resolution of spatial translation along the axial and lateral directions of 100 and 200  μm. The similarity of the profile at this depth was confirmed by numerical simulation using the finite element method with the simulation tool (PZFlex 1.25.3.0, Onscale).

### 2.3 Mice

All mice were housed with littermates in groups of two to five in a reverse day/night cycle and provided ad libitum access to food and water. Male or female C57BL6J wild-type mice aged 8 to 16 weeks (Orient Bio) were used to image the cortical vasculature. Male or female Gad2-GCaMP6-tdTomato mice aged 8 to 16 weeks were obtained by crossing Gad2-IRES-Cre (010802, The Jackson Laboratory) and CAG-floxed-GCaMP6f-tdTomato (031968, The Jackson Laboratory). All animal experiments were performed in compliance with institutional guidelines and were approved by the Subcommittee on Research Animal Care of Seoul National University.

### 2.4 Surgical Implantation of the AOW

The AOW was implanted over the somatosensory cortex following the procedures previously described for the cranial window model.^18^ A mouse received an intraperitoneal injection of dexamethasone (1  mg/kg) 2 h before surgery. After anesthetizing the mouse with isoflurane (induction: 5%, maintenance: 1% to 1.5%, $O_{2}$ at 1  L/min), the mouse was affixed to a stereotaxic frame. The body temperature was maintained at 37°C using a homeothermic blanket. After removing the scalp and periosteum, the cranium over the somatosensory area was removed using a dental drill (diameter ∼4  mm) and the AOW was attached to the exposed cortex with the side of the ultrasound transducer facing the cortex. A customized metal frame was glued around the implanted AOW using a dental cement [Fig. 3(c)]. After the frame was loaded, the MMCX and coaxial cable were firmly fixed to the back of the mouse neck using dermal tape (1527-1, 3M) to relieve the weight of the MMCX connector (0.25 g). The mouse had an acute recovery time of 2 h without receiving anesthetic gas.^19^

### 2.5 *In Vivo* Imaging

The imaging procedure was initiated 2 h after AOW implantation under isoflurane anesthesia. For the vascular imaging, a wild-type mouse received retro-orbital injection of tetramethylrhodamine isothiocyanate-dextran (2.5% w/v, 100  μL in saline; 52194, Sigma-Aldrich). For the imaging of ultrasound-evoked neuromodulation, the AOW implanted on a Gad2-GCaMP6-tdTomato mouse was hooked up to the control board^20^ including a function generator and a power supply [Fig. 3(a)]. The ultrasound parameters used were as follows: frequency, 10 MHz; duty cycle, 50%; acoustic pressure, 0.035 MPa; and stimulation duration, 1 to 20 s. Imaging was performed on a galvanomirror-based multiphoton microscope (Bergamo II, Thorlabs) coupled to a 920-nm femtosecond fiber laser (FemtoFiber Ultra 920, Toptica Photonics). The imaging power was maintained at <60  mW at the objective back aperture. For volumetric imaging, an image stack of $413\times 413\times 200\;\mu m^{3}$ was acquired with 1024×1024  pixels, at an axial interval of 1.5  μm. For functional imaging, time-series imaging of a $413\times 413\;\mu m^{2}$ area was acquired with 256×256  pixels at ∼4  Hz.

### 2.6 Data Analyses

Transmittance of the AOW was acquired using a fiber-coupled spectrometer (CCS200, Thorlabs) and high-power LED (SOLIS-3C, Thorlabs). Two-photon microscopy images were processed using ImageJ FIJI (NIH) and MATLAB (MathWorks). Image processing included adjustment of brightness and contrast, averaged projection of z-stacked images, and time-series analysis of neuronal calcium activity. The calcium activity of F was calculated by dividing the GCaMP6 fluorescence by the tdTomato fluorescence signal. To calculate ΔF, the resting calcium activity during the 10 s prior to stimulation was averaged, and the averaged value was subtracted from the time-series fluorescent signal. The ultimate calcium signal level change of ΔF/F was calculated by dividing ΔF by F. The area under the curve was quantified by integrating the positive area of ΔF/F for 40 s after the start of stimulation, because all the excited calcium signals returned to baseline before the period.

### 2.7 Statistical Analysis

Statistical and regression analyses were performed using the Prism software (GraphPad). Data are expressed as mean ± Standard Error of the Mean (SEM), unless otherwise indicated. A one-way ANOVA (Dunnett’s multiple comparison test) is shown in Fig. 4(c). Statistical significance was set at p<0.05.

## 3 Results

### 3.1 Fabrication of the AOW

To fabricate an AOW capable of being implanted on mouse cranium, we formed transparent acoustic stacks on a circular glass coverslip with a diameter of ∼5  mm [Fig. 1(a), refer to Sec. 2.1 for detailed procedures]. We chose PVDF-TrFE^21^^–^^23^ as the piezoelectric material because it showed sufficient piezoelectricity ($d_{33}=25\;\mathrm{pC}/N$^21^) to generate the 10-MHz signal to stimulate brain cells while being thin enough to achieve decent optical transparency to determine neuronal activities *in vivo*. Considering the small form factor of the glass coverslip, we designed the transducer with an area of $3\times 3\;{\mathrm{mm}}^{2}$. To obtain both optical transparency and acoustic output, we set the thickness of the PVDF-TrFE to 16  μm thick layer of parylene, which provided the best impedance matching the operational frequency (acoustic impedance of 2.5 MRayl^24^) and waterproof characteristics.

As shown in Fig. 1(b), the active area of the AOW appeared to be translucent with a faint yellowish color. To quantitatively investigate the optical transparency, we measured the optical transmittance using a spectrometer. Transmittance was ∼40% at 500 to 1000 nm [Fig. 1(d)]. In two-photon fluorescence imaging, an estimated 60% of attenuation for excitation (920 nm) and emission (546 nm) was compensated by increasing excitation power by approximately fourfold (2.5-fold for compensating two-photon excitation loss and additional 1.6-fold for compensating emission loss).

### 3.2 Acoustic Characterization of the AOW

**Fig. 2:**
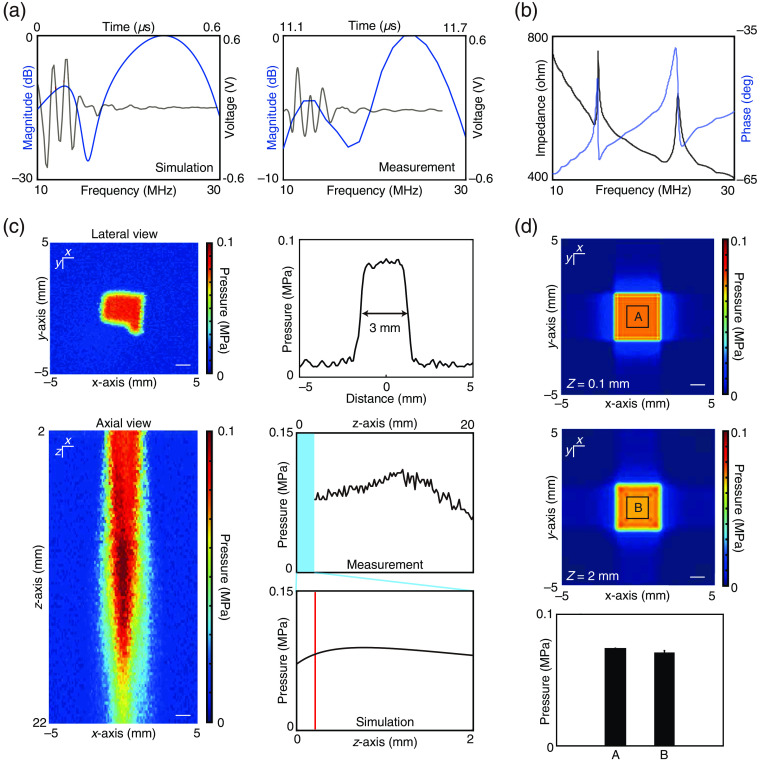
Acoustic characterization of the AOW. (a) The left panel displays the simulated pulse-echo impulse response result in the time and frequency domains in the 170-μm-thick glass coverslip. The right panel displays the measured pulse-echo impulse response result. (b) Measured impedance graph of the AOW. (c) Measured ultrasound beam profiles and pressure line plots in lateral (XY) and axial (XZ) views. Simulated pressure line plot in axial view (XZ) was visualized with a red solid line positioned 100  μm from the surface. The scale bar in XY and XZ is 1 mm. (d) Simulated ultrasound beam profile and averaged pressure in regions A and B in the lateral view with a 2-mm depth and 0.1-mm depth from the surface of AOW. Scale bar, 1 mm.

To characterize the acoustic performance of AOW, we first simulated the pulse-echo impulse response [center frequency of 24 MHz with −6  dB bandwidth of 35.5%; Fig. 2(a)] in glass coverslips with a mean thickness of 170  μm coverslip detected a pulse-echo signal in both the time and frequency domains [center frequency and bandwidth were 24 MHz and 40%, respectively; Fig. 2(a)]. As predicted by the simulation results, a hump in the frequency domain at 17 MHz was also observed in the measured data. In the pulse-echo measurement, the lowest −6  dB frequency was ∼10  MHz, which might be the lowest frequency used for stimulating the brain with the proposed device.

As shown in the impedance curve in Fig. 2(b), there were two resonance peaks in the magnitude curve with corresponding two peak frequencies at 14.9 and 23.5 MHz. Although the impedance of the device at 10 MHz was 750  Ω, which could cause impedance mismatch with the amplifier, the pressure generated from the device at 10 MHz was high enough to stimulate mouse brain.

Because the use of higher frequencies (>1  MHz) could limit ultrasonic wave penetration, we measured the acoustic beam profile of the AOW by scanning the acoustic field using a needle hydrophone installed in a three-axes motorized stage. We measured the pressure with three cycles of 10 MHz with $60\;V_{\mathrm{pp}}$ from 2 to 22 mm axially and laterally [Fig. 2(c)]. The maximum pressure in axial direction was 0.11 MPa at a natural focus depth of 12 mm and measured average pressure in lateral direction at 2 mm was 0.09±0.0009  MPa (standard deviation), corresponding to 1% of the mean value, which might be negligible changes for delivering ultrasound into tissue. Considering the optical penetration depth (<2  mm), we measured the pressure distribution within 2 mm. However, 2 mm was the closest approachable distance to the hydrophone. To overcome this limitation, we performed the PZFlex simulation of the pressure at 0.1 and 2 mm to confirm that there was a negligible difference in pressure between these pressures [Fig. 2(d)]. According to the numerical simulation shown in Fig. 2(d), we inferred that the pressure within an optical accessible depth might be ∼0.078  MPa at 0.1 mm, which was close to the pressure level of 0.075 MPa at 2 mm.

### 3.3 AOW-Based Cranial Window Model

**Fig. 3:**
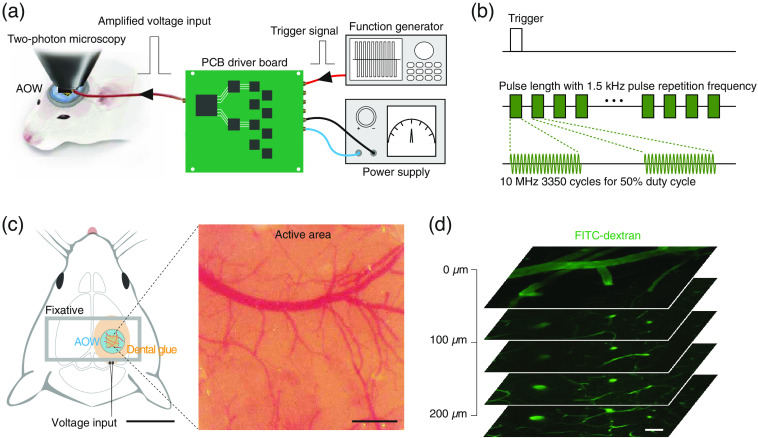
AOW-based cranial window model. (a) Schematic illustration of the overall setup. The AOW is implanted after open craniotomy and connected to the printed circuit board (PCB) driver board fed by a function generator and a DC power supply. The PCB driver board amplifies the input trigger signal by approximately twofold. (b) A schematic illustration of the ultrasound parameter used in the experiment. Frequency, PRF, duty cycle, and pulse length are 10 MHz, 1.5 kHz, 50%, and 1 to 20 s, respectively. (c) Illurstration presenting the structural configurations of AOW-based cranial window implant *in vivo* (left), and a bright field image of the cortical surface visualized through the implanted AOW transducer (right). Note that the scale bars in the left and right figures indicate 10 and 0.5 mm, respectively. (d) A representative z-stacked images of cortical blood vessels up to 200  μm from the pia. Scale bar, 40  μm.

For *in vivo* applications, we implanted the AOW in the mouse brain following a well-established open cranial window preparation.^18^ To generate ultrasound during *in vivo* imaging, a customized ultrasound transmitter^20^ was used to generate patterned stimulation [Figs. 3(a) and 3(b)] with the trigger signal generated by an arbitrary waveform generator (AFG3252, Tektronix). The overall schematic of AOW implantation is shown in Fig. 3(a). After implantation, the cortical surfaces of the pial blood vessels were clearly visible [Fig. 3(c)]. To evaluate the compatibility of the AOW with two-photon microscopic imaging, we imaged the cortical vasculature from the pial surface to 200  μm *in vivo* using Fluorescein Isothiocyanate (FITC)-dextran [Fig. 3(d)]. Owing to the light attenuation of AOW [Fig. 1(d)], the input light power was increased to 60 mW, which had little possibility of a photothermal effect.^25^

### 3.4 Microscopic Observation of Ultrasound-Mediated Neuromodulation *In Vivo*

**Fig. 4:**
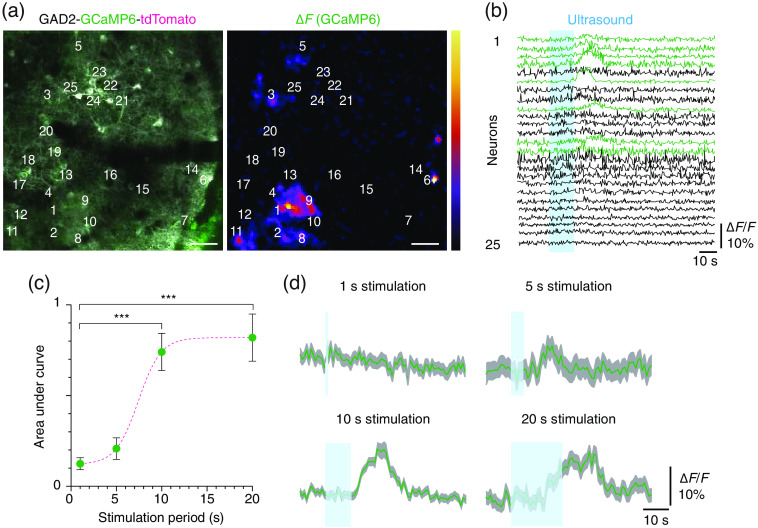
Microscopic observation of ultrasound-mediated neuromodulation using the AOW-based cranial window model. (a) A representative two-photon fluorescence image on the somatosensory cortex of a Gad2-GCaMP6-tdTomato mouse (left) and a pseudocolored image of the change in GCaMP6 fluorescence (ΔF) by the ultrasound stimulation (right). Scale bars, 15  μm. (b) GCaMP6-based neuronal ${\mathrm{Ca}}^{2+}$ traces represented as ΔF/F (n=25 neurons). Ultrasound stimulation was exerted through the AOW during the blue shaded region (input voltage: 20 V). (c) The integrated neuronal activation in response to varying stimulation periods from 1 to 20 s (n=34, 17, 84, and 48 neurons for 1, 5, 10, and 20 s, respectively; green dot: mean, whisker: SEM, acquired from three mice). The dotted curve is fitted to the sigmoidal curve ($R^{2}=0.11$). ***, p<0.0001 (one-way ANOVA). (d) Average ${\mathrm{Ca}}^{2+}$ signal time course of PV neurons in response to varying stimulation periods in (c) (green line: mean, gray shade: SEM, acquired from three mice).

Finally, we tested whether the AOW allows the microscopic observation of ultrasound-mediated neuromodulation. We used a Gad2-GCaMP6-tdTomato mouse line to measure functional calcium activity in a subset of inhibitory interneurons. Two-photon imaging through AOW provided near-artifact-free microscopic imaging of subcellular neuronal structures in Gad2-GCaMP6-tdTomato mice [Fig. 4(a)].

The relatively bright tdTomato signal helped us to confirm the minimal influence of movement artifacts from ultrasonic waves or physiologic motions. Upon ultrasound stimulation at an operation frequency of 10 MHz, a subset of fluorescent interneurons showed a reliable increase in calcium signal with the 10-s stimulation [Fig. 4(b)]. To further test the effect of stimulus duration on neuronal activity, we varied the stimulation period from 1 to 20 s and measured integrated neuronal activity of all neurons detected in different fields of view from three mice [Figs. 4(c) and 4(d)]. With increasing stimulation period, the integrated neural response increased in a sigmoidal relationship. Statistically significant activity was observed for stimulation periods longer than 10 s. During the increasing stimulation period, no significant temperature rise (<0.1°C) was measured by the thermometer with tissue phantom at the focus region. These results suggest that the AOW can be used to screen the influence of ultrasonic parameters on neuronal ensemble activity.

## 4 Discussion

There is an increasing need for two-photon microscopic imaging in ultrasound neuromodulation investigations of neuronal functions *in vivo*. However, the simultaneous monitoring of neuronal responses with ultrasound stimulation is hindered by the conventional transducers and the glass coverslip used in a cranial window model, which increases the attenuation of ultrasonic waves. To apply ultrasound stimulation at the same position as the optical field of view, the stimulant transducer needs to be positioned to introduce an acoustic beam between the optical lens and cortical surface. We presently observed that the incidence of lower frequency along the normal direction reduced the postglass intensity by only 27% and that the ultrasound beam intensity introduced at an angle of 45 deg was reduced by 97% in the postglass region (Fig. S2 in the Supplemental Material). Therefore, a transparent transducer loaded directly on a glass coverslip is necessary for studies that simultaneously combine confocal microscopy monitoring with ultrasound brain stimulation.

Presently, we describe the AOW, an optically transparent acoustic transducer that can be implanted into the mouse brain under anesthesia for monitoring ultrasound-mediated neuromodulation with microscopic precision. An optical transmittance of ∼40% in visible and near-infrared spectral regions was sufficient to image subcellular structures and functional calcium activity across the cortex. An integrated ultrasound transducer has neuromodulatory effects. Considering the versatile use of two-photon microscopy in neuroscience, our AOW could be used as a platform to screen the effects of ultrasound stimuli on neuronal or glial activity.

For more generalized applications, several technical improvements in AOW remain to be addressed. The current version of AOW provides a limited tuning range for ultrasound parameters, such as the frequency and input voltage range. The operational frequency can be further lowered by changing the thickness of the glass coverslip and PVDF-TrFE.^26^ As the simulation results with PiezoCAD in Fig. 2(a) show, the peak frequency of the transducer was lowered from 23.9 to 20.5 MHz with a glass thickness of a quarter-wavelength of 10 MHz compared with the use of half-wavelength of the current glass thickness. However, the method is still limited in further reducing the center frequency to <1  MHz. A thicker PVDF-TrFE can reduce the center frequency of the transducer to 1 MHz, as shown in the simulation results in Fig. S1 in the Supplemental Material. The opacity of the piezoelectric could be increased with an increase thickness because of the inherent low transparency of the PVDF-TrFE <60%. Although the transmittance reportedly increased by quenching the PVDF film, further investigations are needed to develop the fabrication process for AOW.^27^ In addition, electrical impedance matching by elongating the cable or adding a series inductor might increase the sensitivity of the transducer at a lower frequency; however, the size increase of the transducer lowering the electrical impedance is limited by the cranial window. Because of these limitations, our AOW-generating ultrasonic neuronal stimulus parameter was different from that in most previous studies (<1  MHz with <1  s duration^12^^,^^28^ versus 10 MHz with >10  s duration^29^). However, several groups have documented the success of ultrasound in evoking neuronal modulation at >10  MHz for tens of seconds.^29^ Because more studies are required to understand how ultrasound stimuli at different frequencies and durations mediate neuromodulation, the AOW has the potential to provide more comprehensive ultrasound-mediated neuromodulation through the development of advanced piezoelectric materials. A single-crystal piezoelectric material, such as lead magnesium niobate-lead titanate (PMN-PT),^30^ might be an alternative solution because the material has a high dielectric constant, allowing the reduction of the transducer size. However, the compatibility of the material with confocal microscopy needs to be investigated because of its periodic crystal structures.

Unlike the instantaneous excitatory responses to the short ultrasound stimulations <1  s shown in previous studies, this study using stimulations exceeding 5 s resulted in a longer time for ΔF/F to reach from the start of the stimulation to the response peak [Fig. 4(d)]. The delayed response was clearly caused by ultrasound stimulation because the dose-response curve in Fig. 4(c) conformed to the Hill equation, which is mainly evident in biological response curves.^31^ Our dose-response results were similar to those of a previous ultrasound neuromodulation study^12^ and other neuromodulation tools.^32^^,^^33^ A previous study that stimulated inhibitory pathways using high-frequency longer stimulations^29^ identified a poststimulant delay. Although further mechanistic studies are needed to determine the delay time in inhibitory responses with longer ultrasound stimulations, some reports might explain the delayed responses. In addition to the direct stimulation of neurons causing a fast response time, the induction of indirect neuronal excitations related to the astrocytic release of glutamate causing the activation of neuronal N-Methyl-D-Aspartate Receptor (NMDAr) has been described.^34^ Moreover, inhibitory effects with longer time and high-frequency stimulation could induce rebound excitations, which inherently induce delays before acquiring excitatory responses.^35^^,^^36^

Finally, the compatibility of the AOW with optical imaging allows the monitoring of neuronal activity in response to ultrasound stimulation. The fusion of advanced imaging technologies with the AOW would help understand the extensive effect of ultrasound on neuromodulation, as the performance was demonstrated using two-photon microscopy. Although two-photon microscopy allowed the monitoring of neuronal activities from a single in-plane imaging slice, the activities from out-of-focus planes could not be acquired. Robust volumetric imaging techniques, such as the Bessel-beam^37^^,^^38^ or SCAPE,^39^ would help capture the neuronal activities from larger three-dimensional spaces, and the network-level neuronal responses to the stimulation could be investigated.

## Code, Data, and Materials Availability

Custom software scripts and other materials used in this study are available upon request.
