# Supplementary material for: Implantable acousto-optic window for monitoring ultrasound-mediated neuromodulation in vivo
Source: Neurophotonics. 2022 Jul 20;9(3):032203. doi: 10.1117/1.NPh.9.3.032203 (PMC9298854; doi:10.1117/1.NPh.9.3.032203)
Supplement: Supplementary file 1 [file NPh_009_032203_SD001.pdf]

Supplemental Material

Supplemental Figure S1.

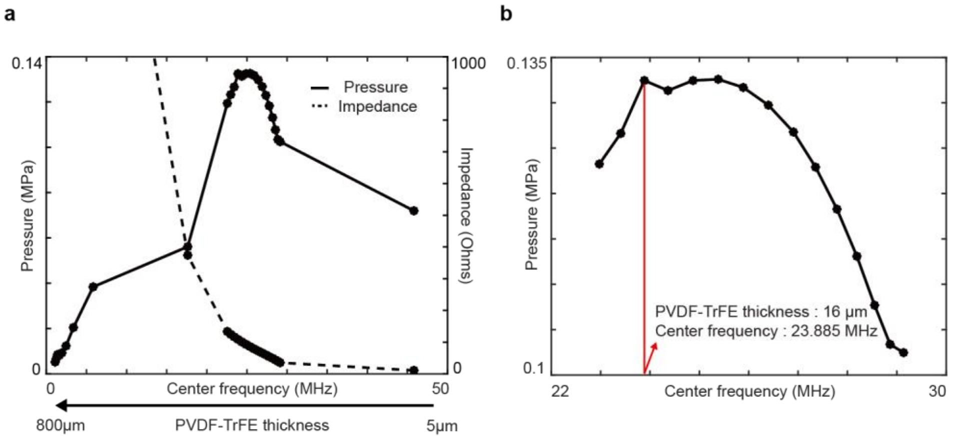

Supplemental Figure 1: PiezoCAD simulation results showing the changes in the output pressure level and electrical impedance of the AOW as a function of frequency. (a) The solid and dashed lines indicate the pressure and electrical impedance levels as functions of the center frequency range from 0 to 50 MHz, respectively. (b) Simulated pressure graph frequency ranges from 22 to 30 MHz. From 24 MHz, which has a PVDF-TrFE thickness of 16 μm, the maximum acoustic output that can be generated.

Supplemental Figure S2.

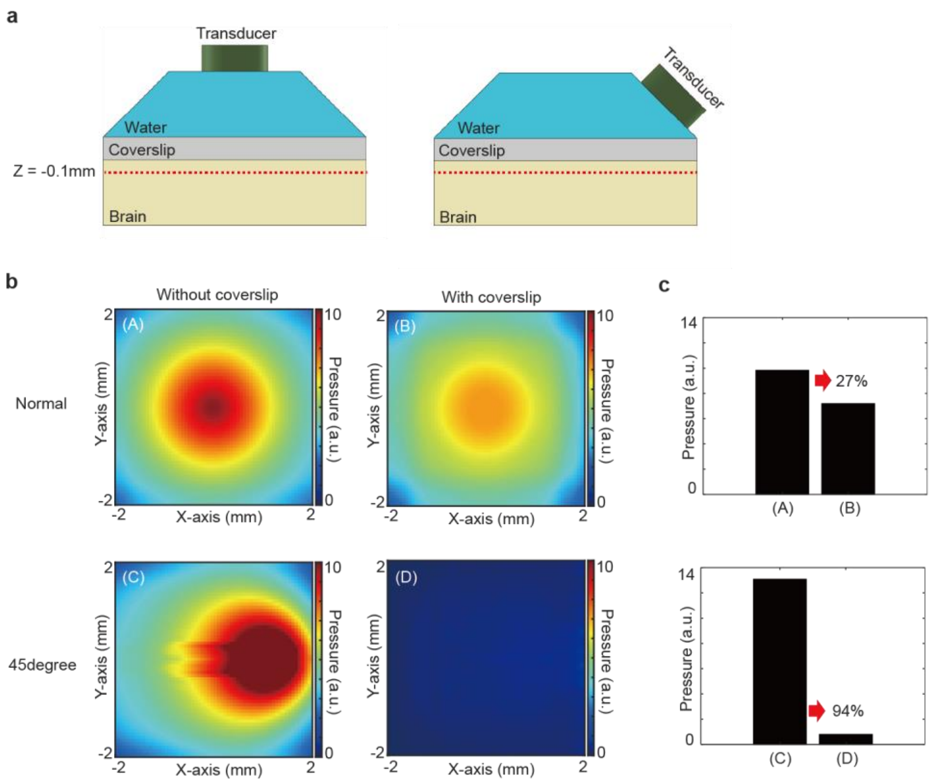

Supplemental Figure 2: PZflex simulation results indicating the extent of 1 MHz frequency ultrasound pressure attenuation with respect to the tilting position of the transducer and the presence of a glass coverslip. (a) Simulation model description with two types of transducer positions. The dashed red line indicates the layer in which the ultrasound pressure was calculated. (b) Simulated ultrasound pressure distribution in the brain tissue layer specified by the dashed red line. (c) Quantified pressure attenuation regarding the presence of a glass coverslip with nominal and 45° tilted transducer positions.
